# A Demonstration Study of the *Quiet Time* Transcendental Meditation Program

**DOI:** 10.3389/fpsyg.2021.765158

**Published:** 2022-01-24

**Authors:** Gabriella Conti, Orla Doyle, Pasco Fearon, Veruska Oppedisano

**Affiliations:** ^1^Department of Economics and UCL Social Research Institute, University College London, London, United Kingdom; ^2^School of Economics & Geary Institute for Public Policy, University College Dublin, Dublin, Ireland; ^3^Research Department of Clinical, Educational and Health Psychology, University College London, London, United Kingdom; ^4^School of Organisations, Economy and Society, University of Westminster, London, United Kingdom

**Keywords:** children, pre-adolescent, transcendental meditation (TM), executive function, socio-emotional skills

## Abstract

This manuscript presents a demonstration study of *Quiet Time* (QT), a classroom-based Transcendental Meditation intervention. The aim of the study is to assess the feasibility of implementing and evaluating QT in two pilot settings in the United Kingdom and Ireland. This study contributes to the field by targeting middle childhood, testing efficiency in two settings operating under different educational systems, and including a large array of measures. First, teacher and pupil engagement with QT was assessed. Second, the feasibility of using a quasi-experimental design and a wide range of instruments to measure changes in pupil outcomes before and after the intervention was assessed. This allows us to obtain information about which instruments might be feasible to administer and most sensitive to change. The first setting included 89 students from a primary school in the United Kingdom: those in sixth grade received the QT intervention, while those in fifth grade practiced meditation using the Headspace application. The second setting included 100 fifth- and sixth-grade students from two schools in Ireland: one received the QT intervention, the other served as a control. Recruitment and retention rates were high in both settings, and the intervention was feasible and accepted by students, parents and teachers. Implementation fidelity was lower in the United Kingdom setting where delivery started later in the school year and the practice was affected by preparation for the Standard Assessment Tests. These results show that QT may be feasibly delivered in school settings, and suggest the use of a compact battery of tests to measure impact. We find suggestive evidence that the intervention affected executive function as children who practiced QT showed improved working memory in both settings. In the Irish setting, pupils in the QT group had improved ability to control responses. These results have implications for future studies by a) demonstrating that implementation fidelity is highly context dependent and b) providing suggestive evidence of the malleability of children’s skills in middle childhood. The results of this demonstration study will be used to inform a larger RCT of the QT intervention.

## Introduction

A recent interdisciplinary literature has focused on the importance of developing social, emotional and executive function skills to promote well-being across the life-course (e.g., Conti and Heckman, 2014). Such skills have been associated with a variety of positive outcomes in adulthood, including improved physical health, better schooling, greater wealth and financial stability, and reduced criminality, risky behavior, and substance use (Heckman et al., 2006; Conti et al., 2010; Moffitt et al., 2011; Heckman and Kautz, 2013). Identifying cost-effective interventions to boost socio-emotional development is particularly important at a time when between 10 to 20 percent of children and adolescents globally experience mental health difficulties (Kieling et al., 2011). Early intervention and prevention are thus an important policy priority (Allen, 2011), which has become even more pressing due to the challenges caused by the COVID-19 pandemic.^1^ The aim of this study is to test the feasibility of implementing one such intervention in schools – transcendental meditation.

### Meditation in Schools

Recently, meditation has experienced growing popularity and interest as a form of school-based intervention to develop habits of the mind and support well-being.^2^ Meditation is considered an attractive tool as it fosters generalizable psychological processes that are crucial for the development of both cognitive and non-cognitive skills (Tang et al., 2007; Travis et al., 2009). The theory of change draws on evidence showing that meditation may create changes in the brain which impact on cognitive functioning, as well as emotional and behavioral regulation. Current evidence indicates that meditation practices are correlated with better self-regulation and emotional stability and less anxiety, reactivity, and risky behavior amongst adults (Eppley et al., 1989; Brown and Ryan, 2003; Tang et al., 2007; Travis et al., 2009; Semple, 2010).

Waters et al. (2015) propose a theoretical model of meditation programs in schools which impact child well-being, social competences, and academic achievement through the enhancement of cognitive functions and emotional regulation. Some schools use popular apps such as Calm and Headspace to introduce meditation into their lesson plans. However, the delivery of meditation via apps usually relies on untrained instructors, so the quality of delivery may vary across different teachers on the basis of their personal involvement with the practice. In contrast, active meditation interventions within the classroom are typically standardized and thus delivered homogeneously across different school settings.

The two main types of meditation interventions offered in schools are Mindfulness Meditation (MM) and Transcendental Meditation (TM). Mindfulness Meditation teaches the ability to direct one’s attention to experience it as it unfolds, moment by moment, with open-minded curiosity and acceptance (Kabat-Zinn, 1990). School-based MM programs use simple techniques designed to enhance mindful awareness of the senses, emotions and behavior, self-regulation, and goal-setting (Greenberg and Harris, 2012). Transcendental Meditation involves the use of a sound or mantra to effortlessly allow the mind to settle down to a state of inner calm. The repetition of the mantra, which is a short word or sound, allows one to reach a state of effortless awareness without concentration or contemplation. The practice does not require any religious belief, philosophy, or change in lifestyle. The practice was popularized in the United States by Mharishi Mahesh Yogi in the 1950’s and differs from other meditation practices as it involves transcending thoughts rather than thinking in the present moment. TM has both psychological and neurophysiological effects. Evidence from studies on adults shows that practicing TM positively impacts brain functioning by promoting higher frontal electroencephalographic coherence and brain integration (Travis and Arenander, 2006), which supports attention, learning, planning, working memory, moral reasoning and emotions. TM is also associated with improved physiological markers of stress (e.g., slower heartbeat, lower blood cortisol levels) (e.g., Pascoe et al., 2017) and cardiovascular risk factors (e.g., lower heart rate and blood pressure) (Levine et al., 2017).

In particular, meditation programs are being implemented in schools to help children improve their socio-emotional skills. A substantive body of evidence indicates that school-based social and emotional skills-based interventions can produce long-term benefits (Durlak et al., 2011; Weare and Nind, 2011). Schools play an important role in raising healthy children as they have regular contact with large numbers of peers across their formative developmental years where lifelong habits are established.

A number of studies have assessed the feasibility and acceptability of implementing meditation programs in schools. In general, these studies have demonstrated mixed results which may be attributed to differences in the content, delivery, and training requirements of the interventions, as well as methodological differences. A systematic review of 31 school-based mindfulness programs by Emerson et al. (2020), concludes that the feasibility of such programs has not yet been established in school settings, that intervention fidelity was achieved in only 45 percent of studies, and standards of teacher training was achieved in only 26 percent of studies. However, inconsistency in the reporting of implementation and fidelity practices hampers this literature.

In addition to implementation studies, a number of studies have investigated the quantitative impact of meditation on children’s outcomes. A systematic review of 15 studies by Waters et al. (2015), examining well-being, social competence, emotional regulation, cognitive functioning, and academic achievement, found statistically significant effects on 61 percent of the outcomes considered. In particular, there were significant effects on 59 percent of the well-being outcomes, 33 percent of the social competence outcomes, 41 percent of emotional regulation outcomes, and 73 percent of the cognitive functioning outcomes. There were too few studies examining the impact of meditation on academic achievement to be considered. The authors also found that TM had a higher proportion of significant effects than MM and other meditation practices (e.g., learning to breathe and attention academy program). The review included a range of experimental, quasi-experimental, non-experimental, and qualitative studies.

More specifically, five TM studies were included in the Waters et al. (2015) review, two of which were specifically based on Quiet Time, the intervention considered here. For example, based on a non-randomized experiment in the US, Nidich et al. (2011), found improvements in English (ES = 0.44) and Math (ES = 0.85) scores among sixth and seventh grade at-risk students who practiced two daily QT sessions; in contrast, an older smaller study by Nidich and Nidich (1989), using a within-group comparison design, found no effects on the academic achievement of 9–17 years old children. While not specifically based on QT, an RCT by So and Orme-Johnson (2001) tested the hypothesis that regular practice of TM for 15–20 min twice a day for 6 to 12 months would improve children’s cognitive ability and well-being. The study included 362 high school students aged 14–18 in three different schools in Taiwan, randomized between treatment and control group. The results showed that TM improved performance on fluid intelligence, information processing speed, practical intelligence and creativity, with effect sizes ranging from 0.13 to 1.00. There was also an impact on well-being (as measured by state and trait anxiety), with effect sizes ranging from 0.38 to 0.62. Using a quasi-experimental design, Baijal et al. (2011) also found evidence that the practice of TM improved attention (effect sizes 0.33–0.41), which is related to academic achievement, on a sample of 155 students from two public secondary schools in India in the 13–15 age group. Finally, a qualitative study by Rosaen and Benn (2006) in the United States reported that children aged 12–14 who participated in TM “described a new way of being, of becoming more self-reflective, and understanding of others.” (p. 423).

Other school-based studies of QT, not included in the review, include Wendt et al. (2015) who used a pretest-posttest design on a sample of 194 young American adolescents, and found higher resilience and less anxiety associated with QT practice, although no effects on academic outcomes. A recent matched-control study evaluating the QT intervention among 101 sixth grade students in the US, also found effects on social-emotional learning (Valosek et al., 2019). Another United States based matched controlled study also found improvements in high school graduation rates following implementation of QT (Colbert, 2013). Finally, a small pre-post study of United States undergraduate students find some evidence that QT lowers anxiety and stress (Burns et al., 2011).

### The Intervention: Quiet Time

The present study provides a demonstration of one such TM intervention in schools. ‘‘*Quiet Time*’’ (QT) involves the practice of Transcendental Meditation, the use of a sound (mantra) to settle down to a calming state.^3^ QT has been in operation for more than 60 years in multiple countries, and in the United Kingdom, its school-based implementation is supported by the David Lynch Foundation (DLF) United Kingdom. School teachers are trained in QT through four one-hour meditation lessons over four consecutive days, undertaken by qualified TM practitioners who are employed through DLF United Kingdom. The teachers are guided on how to conduct the QT sessions in class. They practice QT for a month at the start of the academic year and then start delivering the QT program to their students. Once learned, students practice QT for 10–15 min at the beginning of the school day, and for another 10–15 min at the end of it, until the end of the academic year. Children who opt out of practising QT sit quietly or read during that period. The QT practitioners support teachers and students in their practice on average once a week during the first 3 months after the delivery of the training and then once a month throughout the academic year.

### Objectives of the Study

The objective of the present study is to assess the feasibility of implementing and evaluating QT. This feasibility study adopts a quasi-experimental design in two pilot settings in the United Kingdom and Ireland, with the ultimate aim of using the results of this demonstration to inform a larger RCT of the QT intervention. This study contributes to the literature in three ways, first by focusing on the implementation of QT in middle childhood (among 10–11-year-olds), second, by studying its implementation in two settings – the United Kingdom and Ireland – in which the program has not yet been evaluated, and third, by assessing the sensitivity to change of a wide battery of outcomes and the feasibility of collecting several outcomes. These are discussed in turn below.

First, we focus on middle childhood as it has recently been recognized as a sensitive period for intervention due to the structural changes and reorganization of the brain which occurs during this period (Blakemore, 2012; Sawyer et al., 2012). While early childhood is important for the development of intelligence and self-regulation (Diamond et al., 2007; Heckman et al., 2013; Conti and Heckman, 2014), middle childhood and adolescence is important for the development of academic skills, social-cognitive behaviors and beliefs, and relationship-based skills (Baltes et al., 2006; Bailey et al., 2017; Alan et al., 2021). Thus, there is evidence that certain skills are more or less malleable at different stages of the life-cycle. We also focus on this period of childhood as according to Almond et al. (2018) there are a dearth of studies focusing on the “missing middle.” Therefore, the aim of this manuscript is to examine the malleability of children’s skills in response to an intervention delivered in middle childhood.

Second, while QT is a widely implemented school-based program in the United States, its implementation in Europe has been limited to date. One notable exception is the ‘‘EUROPE’’ project that piloted the QT program in three European countries (Sweden, Netherlands, and Portugal), targeting schools with minority students or those with disadvantaged or migrant background. The project, which is largely based on qualitative assessments, reports positive effects on both pupil and teacher outcomes.^4^ Thus, our knowledge of the acceptability of QT remains limited, and we lack information regarding the feasibility of conducting a quantitative evaluation of the program in the school setting. One key feature of our study is the inclusion of two countries with notable differences in the educational systems, with Irish students typically outperforming United Kingdom students on standardized tests of reading and maths (OECD, Pisa Database, 2018). Thus, assessing the intervention in both settings may reveal important commonalities and differences regarding the implementation of QT.

A third contribution of this study is to include multiple quantitatively assessed outcome measures capturing social connections and preferences, executive function, mental health and socio-emotional well-being, and academic achievement. This allows us to evaluate the feasibility of collecting different outcomes, as well as to assess which of them may be most sensitive to change. As discussed above, meditation can change the structure of the brain, thus potentially impacting multiple aspects of development. Thus, this study can provide a comprehensive account of the types of skills that may be impacted by meditation in middle childhood. Although our pilots do not have the sample size required to detect significant effects, we are able to assess the feasibility of administering a large battery of tests in schools, and to provide a preliminary estimate of the likely impact of the QT intervention.

The specific research questions we address are: (1) are schools, parents, and students willing to engage with the QT intervention and its evaluation? (2) is it feasible to assess impact across multiple dimensions of children’s skills in schools? (3) is the QT intervention acceptable to teachers, students, and parents? and (4) which outcome measures appear most promising as indicators of QT-related change? The reminder of the manuscript is organized as follows. Section 2 describes the methods. Section 3 addresses each of the research questions. Section 4 concludes.

## Materials and Methods

In our analysis, we treat the two pilots separately. There are two main reasons for that. First, there are differences between the two educational systems and societal norms between the two countries, which can differently affect the implementation of the intervention and its acceptance. Second, the majority of the outcomes and part of demographic controls differ between the two studies. Given differences at baseline along demographic characteristics, we preferred to keep the samples separated and control for the range of demographics available.

### Participants and Setting

**FIGURE 1:**
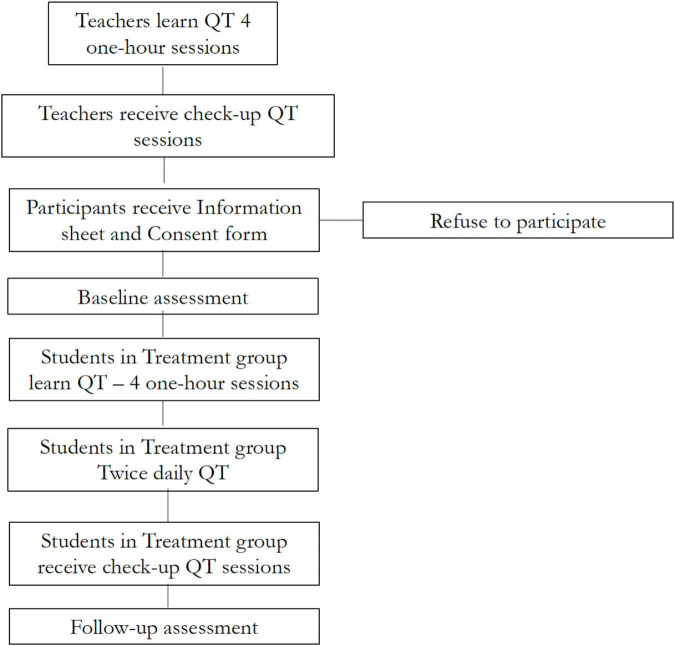
Timeline of the study in both the United Kingdom and the Irish setting.

In the United Kingdom setting, we recruited students from two consecutive grades (fifth and sixth) in a London school.^5^ The minimum age required to practice QT is 10 years. In the United Kingdom, children in fifth grade are between 9 and 10 years old, thus children in sixth grade (age 10–11) were eligible to receive the intervention. Using a quasi-experimental, staggered implementation design, sixth grade students were assigned to the treatment group and learned and practiced QT during the second half of the academic year while fifth-grade students formed the control group^6^ and practiced meditation two to three times a week using the Head Space app. The study ran between December 2018 and July 2019. Figure 1 shows the timeline of the project, which is detailed in this and in the next subsections.

In the Irish setting, we recruited fifth- and sixth-grade students in two primary schools in two adjacent counties (Donegal and Leitrim).^7^ In Ireland, children enter fifth grade when they are 10–11 years old, thus they are eligible to receive the QT intervention. Using a quasi-experimental, staggered implementation design, fifth- and sixth-grade students in the treatment school received the QT intervention, while students in the control school were due to receive the intervention when the study ended. The study started in November 2019 and ended in April 2020 after the COVID-19 outbreak.

In both settings, students who were assigned to learn QT attended a presentation about the research held by two members of the research team and by the TM teacher in their class. During this presentation, the research team talked the students through QT and the study and provided them with a child-friendly information sheet and an assent form, and with an information sheet and consent form for their parents. Neither the treatment nor the control groups received an incentive for participation. The study was approved by the Ethics Review Committee of the Institute of Education, UCL, and the Human Research Ethics Committee in University College Dublin.

### Procedure

In the United Kingdom setting, twelve schoolteachers were trained in QT in November 2018 on the school premises. Six of these teachers were teaching in sixth grade and delivered the QT intervention, the other six were teaching in fifth grade and delivered the Headspace intervention. In the Irish setting, eleven schoolteachers in the treatment school were trained in QT in September 2019. Three of these teachers were teaching fifth and sixth grade and delivered the QT intervention. The training included four sessions over consecutive days (the first session was one-to-one). Follow-up sessions were scheduled once a week for the first 2 months after the delivery of the training and then twice a month until the end of the academic year.

After consent and enrollment, baseline data were collected from all students in December 2018 in the United Kingdom setting and November 2019 in the Irish setting. Questionnaires assessing student outcomes were administered on laptops at school during regular hours. The time for completing the questionnaire was 1 h The QT intervention began in January 2019 in the United Kingdom setting and November 2019 in the Irish setting. In the United Kingdom, the first follow-up was administered 1 week after the students sat their Standard Assessment Tests (SAT) in May 2019. The second follow-up was administered in mid -July before the end of the school year. In the Irish setting, the first follow-up was conducted in March 2020 and parental acceptability was assessed in April 2020. It was not possible to conduct a later follow-up in the Irish setting due to the COVID-19 outbreak.

### Data

#### Implementation Measures

In both settings we included a range of questions to assess program implementation and fidelity. Engagement and compliance with the QT intervention was assessed using a range of measures including recruitment and retention rates and self-reported implementation practices. The acceptability of the QT practice from the perspective of teachers was assessed through teachers’ self-reports and TM practitioners’ self-reports at the check-up sessions. The acceptability of the QT practice from the perspective of the students was assessed at the end of the battery of tests. Students in the treatment groups were asked if they liked QT, if they found meditation easy and if it helped them. If they answered “yes” they were further asked a multiple-choice question to select along which dimensions they felt there was an impact: whether by feeling calmer, by improving relationships within the family, with friends or at school. In the Irish setting we also assessed parental acceptability by asking the parents of treated children in May 2020 (during the COVID-19 lockdown when the schools were closed) if they noticed any change in their child since he/she started to meditate. If they answered yes, they then asked along which dimensions they noticed a difference by selecting one or more of the following options: feeling calmer, less worried, behaving better at home and having better relationships with parents and/or siblings.

#### Outcome Measures

**FIGURE 2:**
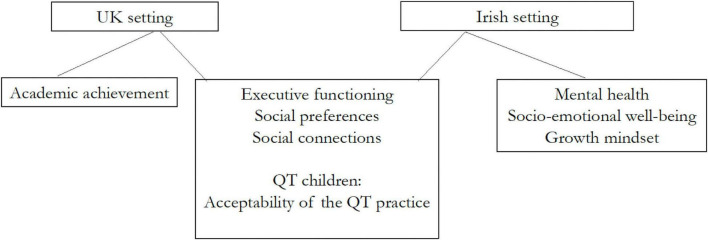
Data collection process.

A key aim of the study Was to assess the feasibility of collecting several outcome measures and their sensitivity to change as result of the QT practice. the outcome measures selected Were motivated by the theory of change presented in Waters et al. (2015). Some additional measures Were collected in the Irish setting, based on learning From the United Kingdom setting. Figure 2 shows the data collection process for the Two settings.

#### United Kingdom Setting

In the United Kingdom setting we collected data on executive functioning, social preferences and connections, and on academic achievement, using the following instruments.

Pupil’s executive function was assessed using two direct assessment measures. The first, the Stroop Color-Word Test, measures the ability to inhibit cognitive interference as the time needed in identifying a color when it is incongruent with the word printed (Stroop, 1935). The pupil is asked to (1) read words that are the names of colors (i.e., word reading), (2) name the color of ink patches (i.e., color naming), and (3) name the color of the ink in which incongruent color words are printed (e.g., say “red” when the word green is printed in red ink). This latter condition is thought to require response inhibition, as one must inhibit the easier and more automatic word reading in order to name the color of the ink (MacLeod and MacDonald, 2000). Children performed ten practice interference trials and, after that, fifty trials over which performance was measured. Performance is measured by completion time.^8^ The effects of response inhibition are indicated by a participant’s slower response times, or minimal target identification when naming the ink color of incongruent color words (interference trial) compared with word reading or color naming. Preliminary evidence indicates that regular meditation practice is associated with an increased ability to focus attention (Chan and Woollacott, 2007; Moore and Malinowski, 2009), but such evidence is not yet available for children or for TM.

The second measure of executive function, Spatial Working Memory (SWM), tests the participant’s ability to retain spatial information and to manipulate remembered items in working memory. It is a self-ordered task, which begins with six phones (boxes) that are shown on the screen. The aim of this test is that, by touching the phone and using the process of elimination, the participant should find the ringing phones in a specific order. There are two sets of this task, one with six phones and one with eight. Performance is measured by the number of mistakes in picking up the correct sequence of ringing phones. A large number of mistakes indicates an inefficient strategy and poorer executive function. Evidence suggests that university students who practice mindfulness perform better than non-practitioners at memory tasks (Zeidan et al., 2010), but there is no such evidence for TM and children specifically.

Social preferences were assessed using a simple form of the dictator game which is a measure of altruistic sharing. One person, the dictator, can unilaterally allocate resources to another anonymous person, the receiver. The receiver cannot reject an allocation offer and cannot punish or reciprocate any action by the dictator. Therefore, if dictators are interested in maximizing their self-gain, they would not offer any resources to the receivers. Consistent with previous studies (e.g., Gummerum et al., 2010), we used the following script to explain the dictator game to the students: “I would like to play a game with you now. This game is called the stickers game. In this game, you can give stickers to yourself and to another child. This child is also a boy or girl and the same age as you. You won’t see the other child and you won’t know who this other child is.” Scores were created by computing the number of stickers which the student agreed to share. The maximum number of stickers to be shared is ten. As sharing resources with strangers constitutes a prototypical aspect of altruistic behavior, a higher number of stickers shared indicates a higher altruistic attitude. Evidence from Galante et al. (2016) indicates that meditation practice increases sharing on a sample of adults, however, such evidence is missing for children and for TM.

Social connections were assessed by eliciting friendship networks: students were asked to name up to five best friends within the same class (Goux et al., 2014). We used the number of nominated friends (“outdegree”) as a measure of social ties. We used the number of friendship nominations received by one student (“indegree”) as a measure of popularity. We also asked the students whether they meet the nominated friends after school, and we used the number of friends met after schools as a measure of the strength of social ties. While the literature has documented the positive role of social ties on education and labor market outcomes (Calvó-Armengol et al., 2009; Conti et al., 2013; Gee et al., 2017), less evidence has been provided on interventions that foster integration in social contexts.

Evidence suggests that academic and non-academic skills such as executive function and social-emotional skills are interconnected and support children’s ability to learn in school (Heckman and Rubinstein, 2001; Best et al., 2011). In addition, as discussed above, there is evidence that while intelligence is largely stable in middle childhood, children’s academic skills are still malleable to intervention. We therefore also collected measures of two academic outcomes: Mathematics and English reading achievement scores. These were obtained from school records at the end of the academic year, and were measured in November 2018 and in June 2019.

#### Irish Setting

The assessments used in the Irish setting were the same as those used in the United Kingdom setting (with the exception of academic achievement which was not available), however, a number of additional instruments were included to capture other dimensions of child development (mental health, socio-emotional well-being, and growth mindset) which may be impacted by the practice of TM.

Student’s mental health and socio-emotional well-being were assessed using a range of instruments. Well-being was measured using the Child Outcome Rating Scale (CORS), a four-item measure designed to assess four areas of life functioning: individual (“How am I doing?”), family (“How are things in my family?”), school (“How am I doing at school?”), and overall well-being (“How is everything going?”). CORS uses child-friendly language and smiling and frowning faces to facilitate the child’s understanding when completing the scales. Ten is the highest score for each of the four scale, with the maximum total score being forty. A total score lower than 32 indicates low well-being. We derive the total score and a dummy variable equal to one if the total score lower than 32, and zero otherwise. Research demonstrates that the CORS has moderate to high reliability and strong concurrent validity with longer, more established measures, with the advantage of being a brief assessment (Duncan et al., 2003; Sparks et al., 2006).

We also administered the Strengths and Difficulties Questionnaire (SDQ), which is widely used to detect mental health problems from childhood through adolescence. The SDQ is a brief questionnaire with 25 items divided into five scales: Emotional symptoms, Behavioral problems, Hyperactivity, Peer relationship problems and Prosocial behaviors. The SDQ follows a Likert response format in which students read a statement and indicate their level of agreement on a three-point scale (not true, somewhat true, certainly true). “Somewhat True” is scored as one, while the scoring of “Not True” and “Certainly True” is zero and two respectively for positive statements, and the reverse for negative statements. The composite Total Difficulties score is generated by summing scores from all the scales except the prosocial scale. The resultant score ranges from zero to 40, and is counted as missing if one of the 4 component scores is missing. Higher scores are associated with more strength in behaviors, emotions and relationships. The “externalizing” score ranges from zero to twenty and is the sum of the conduct and hyperactivity scales. The “internalizing” score ranges from zero to twenty and is the sum of the emotional and peer problems scales. We use the official SDQ cut-off points to compute the fraction of students with high total, externalizing and internalizing difficulties. The SDQ is the most widely used outcome measure of its type in the United Kingdom (Johnston and Gowers, 2005). The measure has sound psychometric properties, with evidence of reliability and validity, when compared with other measures of psychopathology such as the Child Behavior Checklist, the Parent version of the Revised Children’s Manifest Anxiety Scale and the Parent version of the ADHD Questionnaire (Muris et al., 2003).

We also used the Early Adolescent Temperament Questionnaire-Revised (EATQ-R) to assess specific dimensions of socio-emotional well-being including aggression, fear, frustration and inhibitory control (Capaldi and Rothbart, 1992; Ellis and Rothbart, 2001). We selected these dimensions as they are most relevant for the meditation intervention. Subscale scores represent the mean score of all applicable subscale items, scored from one (“Almost always untrue”) to five (“Almost always true”). Higher scores in the aggression subscale indicate a higher propensity toward hostile and aggressive actions and hostile reactivity; in the fear subscale indicate higher unpleasant affect related to anticipation of distress; in the frustration subscale indicate higher negative affect related to interruption of ongoing tasks or goal blocking; and in the inhibitory control subscale a higher capacity to plan and to suppress inappropriate responses. The scale shows meaningful validity when compared with the child version of the Behavioral Inhibition Scale, Revised Child Anxiety and Depression Scale and Child Rating scale for Aggression (Muris and Meesters, 2009).

The last additional outcome was growth mindset, which assesses the belief about how much one can change one’s own intelligence. People with a growth mindset believe that they can become smarter with effort, while those with a fixed mindset believe that they are born with a certain amount of intelligence and there is little they can do to change it. Evidence shows that students with growth mindsets are more likely to enjoy the academic process, and have higher academic achievements and well-being (Aronson et al., 2002; Blackwell et al., 2007). Growth mindset is assessed using three statements ranked on a 6-point scale (1 = strongly agree; 6 = strongly disagree). Students elicit how much they agree with statements such as “You can learn new things, but you can’t really change your basic intelligence” (reversed item). We take the average score over the three items. A higher score indicates a greater growth mindset.

### Analysis Methods

The implementation data is presented using descriptive statistics. The outcome data is presented using descriptive statistics and random effects model. These are explained in more detail below.

## Results

In this section we address the key research questions on engagement, feasibility, and acceptability, and we present a comparison of the outcomes of the QT treatment and control groups.

### Engagement

#### United Kingdom Setting

**FIGURE 3:**
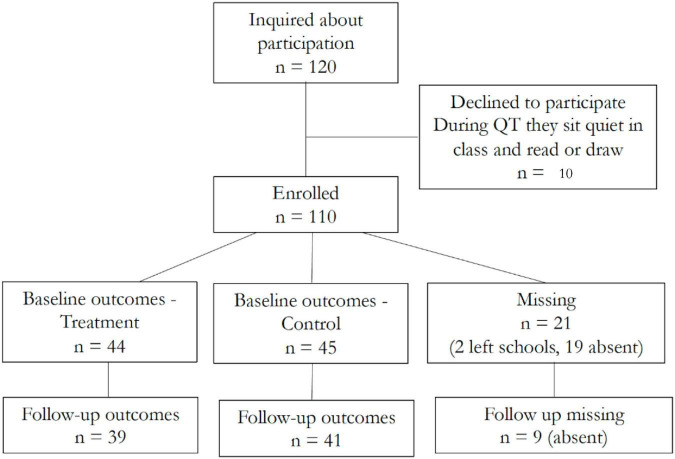
CONSORT flow diagram – United Kingdom setting.

Figure 3 reports the CONSORT flowchart that shows the initial number of students recruited and the number of students for whom full data was gathered in the United Kingdom setting. Of the 120 eligible parent–student pairs (of 450 children in the school), 110 (92%) of parents agreed that their children could participate in the study, with 10 students opting out from the treatment group. Two students left the school during the academic year. In total, 50 sixth-grade students received the QT intervention and 60 fifth-grade students were part of the control group. 89 students participated in the baseline testing; 9 of them were absent at the second follow-up in July (4 from the control group and 5 from the treatment group). This low attrition rate is consistent with the findings of Emerson et al. (2020) who report average retention rates of at least 80% in their systematic review.

Table 1 shows the descriptive statistics for the children in the United Kingdom sample who agreed to participate in the QT intervention and completed the assessment at baseline. It also shows the differences in means between treated and control groups with corresponding *p*-values based on permutation tests.^9^ There are no statistically significant differences between the treatment and control groups across individual characteristics such as ethnicity (white, black, and other ethnicity), free school meal eligibility (FSM, as a proxy for economic and social disadvantage), and English as not the first language (EAL).^10^ The average student’s age is about 10.5 years old. Students in the treatment group are almost 6 months older than those in the control group as expected. Age may be correlated with some of the outcomes of interest, as older students are likely to perform better than younger ones, thus we control for month and year of birth when we compare the outcomes of the treatment and control groups. The proportion of girls is smaller in the control group (38 percent) than in the treatment group (55 percent), but the difference is not statistically significant. 22 percent of the sample is eligible for a free school meal, and about a third of children do not speak English as their first language. These numbers are higher than the national averages: data from the Department for Education (2018) shows that, in primary schools, the proportion of children eligible for free school meal is 13.7 percent and the proportion of children not speaking English as a first language is 21.2 percent.^11^

**TABLE 1:** Descriptive statistics at baseline, United Kingdom setting.

	Grade 5 – Control	Grade 6 – Treated	
	Mean/%	Mean/%	Diff (*p*-value)
Age	10.117 (0.47)	10.84 (0.28)	0.728*** (0.00)
Female %	0.378	0.545	0.168 (0.20)
White %	0.289	0.364	0.075 (0.46)
Black %	0.311	0.200	−0.107 (0.30)
Other – Ethnicity %	0.400	0.430	0.032 (0.85)
Free school meals (FSM) %	0.270	0.180	−0.085 (0.42)
English second language (EAL) %	0.360	0.270	−0.083 (0.54)
Number of obs.	45	44	

*Mean and standard deviation reported in parenthesis for continuous variables. Proportions reported for binary and categorical variables. p-values of the permutation tests are shown in parenthesis next to the Diff column.*

***p < 0.001 refer to difference in means.

Additionally, one third of students in the sample are ethnically White, about one fourth Afro-American or Caribbean (slightly more in the control group), while the others have mixed or another group ethnicity. In general, the treatment group appears older and positively selected under some observable characteristics (although not at a statistically significant level); we control for these observable differences when we compare the outcomes across groups.

#### Irish Setting

**FIGURE 4:**
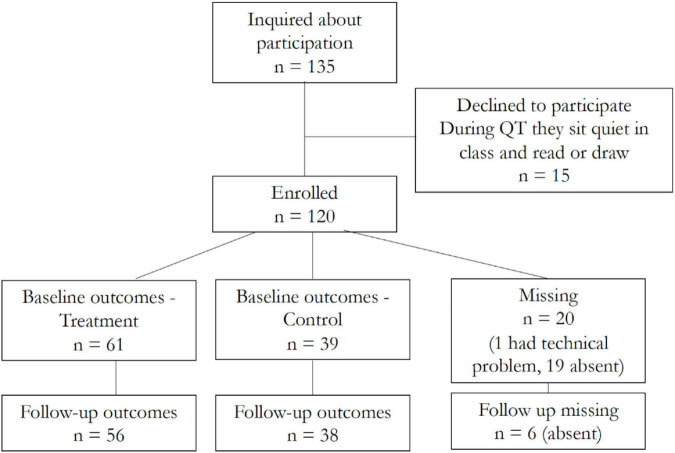
CONSORT flow diagram - Ireland setting.

Figure 4 shows the CONSORT flowchart that reports the number of students recruited and the number of students for whom full data was gathered in the Irish setting. 75 of the parents in the treatment school consented for their children to participate in the study (100 percent), while only 45 of the parents in the control school (76 percent) agreed; this is possibly due to parents not being comfortable with their children being in a control group.^12^ 100 of the 120 students participated in the baseline testing (61 from the treatment school and 39 from the control school). 19 children missed the baseline test because they were absent on that day (14 from the treated school and 5 from the control school), and one had a technical problem with the laptop and was not able to upload the results of the tests. 6 students were absent at the follow-up. The high number of missing students on the assessment day may be driven by relatively high rates of school absenteeism due to illness during this time of year (end of November and beginning of December).

Table 2 shows the descriptive statistics for the Irish sample who agreed to participate in the QT intervention and completed the assessment at baseline. It also shows the differences in means between treated and control groups with corresponding *p*-values based on permutation tests. Slightly less than half of the students are girls and the average age is about 11.6 years old. There are no statistically significant differences across the treatment and control schools by gender, however, a higher fraction of students has a learning disability, such as dyslexia and ADHD, in the control school (20 percent) than in the treatment school (2 percent).^13^ Also, students are significantly older in the control school. We control for all baseline differences when we compare the outcomes of the treatment and control groups.^14^ Other demographics such as ethnicity and socio-economic status were not available.

**TABLE 2:** Descriptive statistics at baseline, Ireland setting.

	Control school	Treatment school	
	Mean	Mean	Diff (*p*-values)
Age	11.66 (0.6)	11.45 (0.57)	−0.212 (0.03)
Female %	0.49	0.41	−0.077 (0.60)
Learning disability %	0.2	0.02	−0.188** (0.00)
Number of observations	39	61	

*Mean and standard deviation reported in parenthesis for continuous variables. Proportions reported for binary and categorical variables. p-values of the permutation tests are shown in parenthesis next to the Diff column.*

**p < 0.01 refer to difference in means.

### Feasibility of Data Collection

This section assesses the feasibility of administering a wide range of tests in schools measuring a set of outcomes that are predicted to be impacted by meditation. We found that not all children completed the full battery of tests in both studies. We checked if the number of tests completed was significantly different for the treatment and control groups but were unable to detect any significant effect.

#### United Kingdom Setting

The students completed the assessments using Psytool, a toolkit that runs cognitive-psychological tasks. They were given 1 h to complete the tests (due to class scheduling requirements), however some were not able to complete the whole battery of tests within that timeframe. The tests were conducted during school hours, with some classes completing them in the morning and others in early afternoon. The data collection process for the first follow-up was scheduled in May 2019. However, the majority of students, possibly due to the stress of performing the SAT the week before, only partially completed the assessment. Another issue that occurred at all data collections points concerned unforeseen technical problems administering the Stroop Color-Word Test and the Working Memory tasks on the school laptops, which were the two tasks which required internet speed and computer efficiency to properly run. This resulted in the following sample losses: 18 percent did not report social connections at follow up; 5 percent did not complete the stickers game at follow up, 16 percent did not complete the Stroop Color-Word Test (both at baseline and follow-up) and 8 percent did not complete the Spatial Working Memory (both at baseline and follow-up). Reassuringly, there was no differential completion between students in the treatment and control groups at follow-up. Additionally, two-thirds of students did not complete the final questionnaire on the acceptability of the intervention at the second follow-up, which was at the end of the battery of tests. Not speaking English as a first language and Afro-American or Caribbean ethnicity were significantly negatively correlated with the probability of completing the acceptability questions.

#### Irish Setting

Similar to the United Kingdom setting, some students in the Irish sample did not complete the full battery of tests within the scheduled time, for two main reasons: the 1-h time constraint and the occurrence of some technical difficulties in administering the Stroop Color-Word Test and the Working Memory on school laptops. As a result, 23 percent (similarly distributed at baseline and follow-up) and 25 percent (30 percent at baseline and 20 percent at follow up) of students did not complete the Stroop Color-Word Test and the Spatial Working Memory. Reassuringly, there was no differential completion between students in the treatment and control groups at follow-up. The other tests, which are based on questionnaires rather than tasks, showed higher completion rates, with less than 5 percent of students not completing at least one of them, similarly distributed at baseline and follow-up. A total of 54 students (88 percent) completed the final questionnaire on the acceptability of the intervention at the second follow-up, a higher fraction than in the United Kingdom setting.

In sum, in both settings the data collection encountered some challenges, particularly in relation to the Stroop Color-Word Test and the Working Memory Task, whose completion was affected by technical issues with the software.

### Teacher Acceptability

#### United Kingdom Setting

In terms of program delivery, QT was regularly practiced by the teachers from January until mid-March 2019. The teachers interrupted the meditation practice 2 months before the first follow-up assessment in May to concentrate on the preparation for the SAT, and they resumed it with regularity afterward. This indicates that the delivery of the intervention needs to be carefully timed over the school year, otherwise lack of planning may affect implementation fidelity.

School teachers were not required to record how frequently they performed the QT practice. Therefore, we cannot assess the proportion of sessions completed. Instead, we relied on the teachers’ reports and the QT practitioners’ check-up sessions to infer intervention fidelity. A possible improvement in future studies could be to use an app where teachers can sign in and track their daily meditation practices, allowing, if used properly, to measure compliance with the treatment and its intensity. For this study, reports by both the schoolteachers and the TM practitioners suggest that the teachers engaged with the technique and practiced it by themselves initially, and that they subsequently implemented QT with the students in the class every day.

The follow-up check-up meditation sessions for the students were scheduled and delivered once a week until the end of March. After the interruption due to the preparation for the SAT, they resumed with the same frequency from the end of May until the end of the school year.

#### Irish Setting

As in the United Kingdom setting, we relied on the schoolteachers’ self-reports and the QT practitioners’ check-up sessions to assess implementation. All teachers in the treated classes engaged with the technique and initially practiced it by themselves. They then started to implement QT with students in class. The follow-up meditation sessions for the students were delivered as planned. Both teachers and QT practitioners reported that the intervention was implemented with fidelity over the academic year until the COVID-19 lockdown on March 12, 2020. As the intervention was delivered earlier in the academic year in the Irish setting compared to the United Kingdom setting, this may have contributed to establishing the habit of regularly practicing QT, leading to its continuity.

### Student/Parent Acceptability

#### United Kingdom Setting

Among those who completed the questions on acceptability, 17 students (59 percent) reported that they found the practice of QT easy, and 11 (38 percent) reported that they liked the practice and found it helpful. Thus, most students (62 percent) did not report finding the practice useful, which may be related to the 2-month gap which occurred during QT implementation due to the SAT preparation.^15^ This is in contrast with the findings reported in Emerson et al. (2020) who found relatively high student satisfaction ratings in terms of enjoyment (∼50–70 percent) and willingness to recommend the program to others (∼89–92 percent) in relation to mindfulness interventions. Among those reporting that the QT practice helped them, 11 (79 percent) said they felt calmer, 4 (29 percent) stated that it helped them at school, 2 (14 percent) with friends, and 1 (7 percent) with their family. The low completion rates suggests that the time allocated for the assessment could be increased or the battery of tests reduced to ensure higher rates of completion.

#### Irish Setting

As in the United Kingdom setting, we assessed the acceptability of the intervention among the students in the follow-up: 57 percent of students completed this section of the questionnaire, which was higher than in the United Kingdom setting. 48 students (76 percent) reported that they found the practice of QT easy and 41 (70 percent) reported that they liked the practice and found it helpful. These figures are more in-line with Emerson et al. (2020) systematic review, as compared to those from the United Kingdom setting. When addressing which dimensions of the QT practice the students felt had helped them, 25 (66 percent) said they felt calmer, 16 (42 percent) stated that it helped them at school, 13 (34 percent) with friends, and 16 (29 percent) with family.

Parental acceptability was assessed in April 2020, however, only 17 of the 75 parents in the treatment group completed the questionnaire, perhaps due to the pressures of home schooling and working during the pandemic. When asked whether their children were practicing meditation during the lockdown, 7 (40 percent) reported that their children meditated every day or 3–5 times a week, 5 (30 percent) once a week, and 5 (30 percent) reported that their children were not practicing at all. When asked whether they noticed any change in their children since they started practising meditation, 12 (70 percent) reported that they did: 10 (60 percent) said that their children seemed calmer, 5 (30 percent) stated that they behaved better at home, 2 (12 percent) that they seemed less worried and 2 (12 percent) that they were having better relationships with their parents/guardians or siblings.

### Sensitivity to Change

The aim of this section is to give an indication as to which of the outcome measures may be most sensitive to change after the QT intervention. The empirical strategy consists of comparing the difference in outcomes before and after the QT intervention for students in the treatment group to the same difference for students in the control group. This methodology is appropriate when the intervention is as good as random, so that the trend in the outcomes for the control group can be a valid comparison and the difference in the trends between the treatment and the control group can be interpreted as the effect of the treatment. In other words, this “difference-in-differences” methodology makes the “parallel trends” assumption (i.e., in absence of the treatment, both the treated and the control group would be on parallel trends). Given the lack of pre-treatment data at multiple timepoints, unfortunately we are unable to check whether this assumption is likely to hold in our case. All our estimates are based on an ‘intention-to-treat’ (ITT) analysis, i.e., we compare the available outcomes of all students allocated to the treatment or to the control condition, regardless of whether or not they actually received the QT intervention.

In the econometric implementation, we use a random effects panel data linear regression model, which takes into account the longitudinal nature of the data by exploiting information on the same participants at multiple points in time. The baseline model includes a binary indicator for being in the treatment group (versus control), a binary indicator for follow-up (versus baseline), and an interaction between the two. A second specification includes individual controls, and the third specification also adds baseline values of the outcome variable and the unbalanced variables at baseline. In all models, standard errors are clustered at the class level to account for intra-cluster correlation.

Missing data for students who did not complete one of the tasks at baseline and follow-up were imputed using multiple imputation, a statistical technique which uses the distribution of observed data to estimate a set of plausible values for the missing data. The missing values are replaced by the estimated plausible values by the estimation of multiple datasets (10 in our case)^16^. The results obtained from each dataset are combined using Rubin’s rules to create a “complete” dataset (Schafer, 1999).

#### United Kingdom Setting

Due to a lack of consistent completion of the assessment in the first follow-up in May 2019 and also due to reduced implementation fidelity before the time of the assessment (because of the SAT preparation), we only focus on data from the baseline and the second follow-up in July 2019. Thus, we analyze the sample of 89 students who completed the baseline and 80 students who completed the follow-up.

Table 3 shows the outcome data for the treatment and control groups at baseline and follow-up. Columns six and eleven report *p*-values from permutation tests testing for differences between the treated and controls groups at both time points. Panel A shows the baseline data for the groups before the QT intervention. There are no statistically significant differences between the groups on any of the baseline outcome measures, except for the Math score, which is 3.73 points significantly higher in the treatment group.^17^ Panel B shows the outcomes for the treatment and control groups after the QT intervention. There are no statistically significant differences between the groups on any of the outcome measures, except for the numbers of errors in the Working Memory task, which is significantly lower in the treatment group (by 2.35 in the 6 tasks version and by 7.79 in the 8 tasks version), and the measure of strength of social ties, which increases and points toward significance in the treatment group. These differences are tested below using the multivariate regression framework.

**TABLE 3:** Descriptive statistics of the outcome variables before and after the intervention in the United Kingdom setting.

	Baseline					Post-treatment				
	Treat	Mean	*SD*	Diff	*N*	Mean	*SD*	Diff	*N*	Max
Reaction time, Stroop	QT	1,580.797	583.23	−51.790	44	1,320.774	664.17	77.338	36	n/a
	Con	1,632.587	653.26		31	1,243.436	325.34		31	
Errors, working memory task, 6	QT	4.068	4.49	−1.774	44	3.056	4.58	−2.350*	36	n/a
	Con	5.842	4.85		38	5.405	4.03		37	
Errors, working memory task, 8	QT	14.545	7.00	−1.323	44	9.94	7.94	−7.785**	36	n/a
	Con	15.868	8.00		38	17.73	8.95		37	
Stickers given – Dictator game	QT	3.14	3.48	−0.508	44	5.237	2.92	1.47	38	10
	Con	3.644	3.98		45	3.76	3.72		38	
Social ties – total	QT	4.70	0.63	0.46	44	4.242	1.82	0.24	33	5
	Con	4.244	1.43		45	4.00	1.90		33	
Social ties – strong friendships	QT	1.34	1.58	0.32	44	2.303	2.04	0.88	33	5
	Con	1.02	1.59		45	1.42	2.02		33	
Social ties – popular	QT	3.95	2.64	0.33	44	3.56	2.71	0.93	39	n/a
	Con	3.62	2.52		45	2.63	2.70		41	
English reading	QT	103.48	4.30	0.26	44	107.20	6.76	1.449	39	120
	Con	103.222	6.13		45	105.756	5.50		41	
Math	QT	109.86	5.78	3.730**	44	108.615	5.33	2.40	39	120
	Con	106.13	6.19		45	106.22	6.83		41	

*QT, treatment group; Con, control group. The baseline was collected in December 2018 and the post-treatment data in July 2019. P-values of the permutation tests are shown in parenthesis in the Diff column.*

**p < 0.01 and *p < 0.05 refer to difference in means.

The results from the random effects (RE) panel data estimation are shown in Table 4, where we report the coefficients of the interaction between the Treatment and Time dummies (standard errors in parenthesis). The first column shows the mean value of the outcome variable to help assess the size of the effects. The other three columns include models which have different sets of control variables: in the first column the controls are time (post- versus pre-intervention), school class and treatment group dummies (sixth grade versus fifth grade), plus the interaction between the time and the treatment group dummy. In the second column we control for age, which was unbalanced at baseline, with months and years of birth dummies. In the third column the controls also include gender, dummies for ethnic group (white, black and other ethnicity), free school meal eligibility and English as not the first language, and a dummy for the test being taken in the morning (before 12 am). The latter is not included for the academic outcomes, that were not assessed in the battery of tests. In the third column we add the baseline value of the outcome variable (which helps to increase the efficiency of the estimates) and the math score at baseline, as it was unbalanced between the treatment and control groups. The fourth column includes all controls and shows the results of the interaction between the treatment status dummy, the post-intervention dummy and a dummy for female to test for gender differences.

**TABLE 4:** Random effects panel estimates – Treatment impacts in the United Kingdom setting.

	Mean		Treat*Time			Treat*Time*Fem.	*N*
	Depvar	RE-1	RE-2	RE-3	RE-4	RE-5	
Reaction time, Stroop	1633	100.432	113.6	163.712	132.850	−135.604	169
		[101.660]	[101.447]	[208.795]	[150.838]	[122.555]	
Errors, working memory task, 6	5.84	−1.622*	−1.096	−3.595*	−3.406	−0.530	169
		[0.721]	[1.277]	[1.673]	[2.024]	[1.143]	
Errors, working memory task, 8	15.86	−6.652**	−5.672**	−7.171*	−6.363*	4.075	169
		[0.513]	[1.018]	[3.302]	[3.402]	[4.667]	
Stickers given – Dictator game	3.64	1.575*	0.975*	1.453	0.518	−0.125	169
		[0.755]	[0.393]	[1.316]	[1.805]	[1.264]	
Social ties – total	4.24	0.009	−0.396	−0.825	−0.902	1.144*	169
		[0.639]	[0.420]	[0.704]	[0.703]	[0.535]	
Social ties – strong friendships	1.02	0.593	0.36	−0.964	−1.286	0.49	169
		[0.476]	[0.324]	[0.894]	[1.125]	[0.251]	
Social ties – popular	3.62	0.584	0.361	−1.895	−2.127*	−0.420	169
		[0.495]	[0.568]	[0.979]	[1.067]	[0.903]	
English reading	103.2	1.186	1.174	1.022	1.197	−0.200	169
		[1.643]	[1.689]	[1.706]	[1.765]	[1.189]	
Math	106.1	−0.810	−1.197	−1.476	−1.444	1.158	169
		[0.916]	[0.905]	[1.048]	[1.047]	[0.595]	

*Robust standard errors clustered at class level in brackets. Controls in model RE-1 include time (July 2019 versus December 2018), treatment (sixth grade versus fifth grade) and class dummies. Controls in model RE-2 additionally include month and year of birth dummies. Controls in model RE-3 additionally include: dummies for gender, FSM, EAL, black and other ethnicity, and a dummy for the test taken in the morning (for all outcomes except the academic ones). Controls in model RE-4 additionally include the baseline value of the dependent variable. Controls in model RE-5 are the same as in model RE-4, and the coefficient shows the interaction with the female dummy. The sample size consists of 89 observations at baseline, and of 80 at the follow up. Missing data are imputed.*

**p < 0.01 and *p < 0.05.

Table 4 shows that there is no significant change associated with the QT intervention for most outcomes (Stroop Test, social connections, and preferences and academic achievement), except for working memory. In this case, the number of errors significantly decreases in the 8 task version of the Working Memory Task at follow-up for the treatment group.^18^ When we look at gender differences, there is a significant improvement in the social network measures for girls in the treatment group, who increased the number of friends in their network by 1 (from a baseline mean of 4). In sum, these findings suggest that QT has a limited impact on student outcomes in the United Kingdom setting.

#### Irish Setting

Table 5 shows the outcome data for the treatment and control groups before and after the QT intervention in the Irish setting. Columns six and eleven report *p*-values from permutation tests testing for differences between the treated and controls groups at both time points. Panel A shows that there are some statistically significant differences for the following outcomes at baseline: the aggression score, which is 0.5 points lower in the treatment group; the inhibition score, which is 0.5 points higher in the treatment group; the total number of friends and the number of friends seen outside school, which are significantly higher by a factor of 1 and 0.5 in the treatment group. Panel B in Table 5 shows the outcomes at follow-up. Again, there are some statistically significant differences for the following outcomes: the aggression score, which is significantly lower in the treatment group; the inhibitory control score and the number of friends seen outside school, which are significantly higher in the treatment group; the measure of popularity, which is significantly higher in the treatment group (for this measure there was no significant difference at baseline). These differences are then formally tested in the multivariate regression analysis.

**TABLE 5:** Descriptive statistics of the outcome variables before and after the intervention in the Irish setting.

	Baseline					Post-treatment				
	Treat	Mean	*SD*	Diff	*N*	Mean	*SD*	Diff	*N*	Max
Child Outcome Rating Scale	QT	33.95	5.30	2.00	61	32.857	5.94	1.91	56	40
	Con	31.95	7.23		39	30.946	7.84		37	
Low child Outcome Rating	QT	0.34	0.48	−0.040	61	0.38	0.49	−0.139	56	1
	Con	0.38	0.49		39	0.514	0.51		37	
High Social Difficulty Score	QT	0.364	0.48	0.066	55	0.315	0.47	−0.010	54	1
	Con	0.297	0.46		37	0.324	0.48		37	
High externalizing score – SDQ	QT	0.109	0.32	0.028	55	0.037	0.19	−0.044	54	1
	Con	0.081	0.28		37	0.081	0.28		37	
High internalizing score – SDQ	QT	0.20	0.40	−0.070	55	0.148	0.36	−0.01	37	1
	Con	0.27	0.45		37	0.162	0.37		37	
Aggression, EATQ	QT	1.733	0.82	−0.463**	60	1.61	0.56	−0.291*	55	5
	Con	2.197	0.73		39	1.90	0.65		38	
Fear, EATQ	QT	2.84	0.85	0.141	60	2.715	0.70	0.06	55	5
	Con	2.70	1.00		39	2.658	1.00		38	
Frustration, EATQ	QT	2.75	0.72	−0.25	60	2.66	0.71	−0.17	55	5
	Con	3.00	0.72		39	2.83	0.71		38	
Inhibitory control, EATQ	QT	3.82	0.75	0.394**	60	3.87	0.71	0.257*	55	5
	Con	3.43	0.54		39	3.62	0.56		38	
Growth mindset	QT	3.21	1.39	−0.23	60	3.47	1.43	0.09	56	6
	Con	3.44	1.33		39	3.38	1.26		38	
Reaction time, Stroop	QT	1387.34	461.76	142.546	53	1282.36	364.09	−91.249	44	n/a
	Con	1244.80	493.98		24	1373.61	568.59		29	
Errors, working memory task, 6	QT	6.40	4.61	2.10	47	4.023	3.77	0.958	44	n/a
	Con	4.30	4.22		23	3.065	3.51		31	
Errors, working memory task, 8	QT	16.45	9.87	2.664	47	15.295	8.86	2.650	44	n/a
	Con	13.78	7.22		23	12.645	9.18		31	
Stickers given – Dictator game	QT	2.32	3.36	−0.28	56	4.019	3.86	1.137	52	10
	Con	2.61	3.44		38	2.882	3.14		34	
Social ties – total	QT	4.78	0.81	0.509*	59	4.839	0.73	0.145	56	5
	Con	4.27	1.48		37	4.694	0.75		36	
Social ties – strong friendships	QT	2.95	1.72	0.976*	59	2.911	1.77	0.827*	56	5
	Con	1.97	1.89		37	2.08	1.48		36	
Social ties – popular	QT	3.95	2.62	0.49	59	4.821	3.11	1.488*	56	n/a
	Con	3.46	2.22		37	3.33	2.01		36	

*QT, treatment group; Con, control group. SDQ, Strength and Difficulties Questionnaire; EATQ, Early Adolescent Temperament Questionnaire. P-values of the permutation tests are shown in parenthesis in the Diff column.*

**p < 0.01 and *p < 0.05 refer to difference in means.

The regression results displayed in Table 6 show the coefficients of the interaction between the treatment (treatment versus control school) and the time (March 2020 versus November 2019) dummies obtained from the random effects panel data estimation. The first column shows the mean value of the outcome variable. The other three columns have different sets of control variables: the first column displays the results of the model where we control for time, school class and treated school dummies, and the interaction between time and treated school. In the second column we also include gender, month and year of birth dummies, a dummy for the test being taken in the morning (before 12 am), a dummy to indicate a learning disability and the interaction between disability and time. In the third column we also control for the baseline value of the outcome variable and for all of the measures where there were differences at baseline: aggression, inhibition, number of friends, and strength of social ties. The last column shows the results of the interaction between treatment status and a dummy for female and includes all controls.

**TABLE 6:** Random effects panel estimates – Treatment effects in the Irish setting.

	Mean	Treat*Time				Treat*Time*Fem.	*N*
	Dep.var.	RE-Model1	RE-Model2	RE-Model3	RE-Model4	RE-Model5	
Child Outcome Rating Scale	31.95	0.139	0.137	−0.848	−0.401	0.095	194
		[0.899]	[0.907]	[1.549]	[1.158]	[0.629]	
Low Child Outcome Rating	0.384	−0.101	−0.101	−0.031	−0.058	0.039	194
		[0.082]	[0.083]	[0.090]	[0.056]	[0.090]	
High Social Difficulty Score	0.297	−0.064	−0.064	−0.089	−0.083	0.083	194
		[0.127]	[0.127]	[0.148]	[0.139]	[0.081]	
High externalizing score – SDQ	0.0811	−0.127	−0.126	−0.176	−0.166	0.032	194
		[0.103]	[0.102]	[0.217]	[0.218]	[0.121]	
High internalizing score – SDQ	0.270	−0.132	−0.13	−0.391	−0.340	−0.184	194
		[0.175]	[0.173]	[0.403]	[0.412]	[0.227]	
Aggression, EATQ	2.197	0.134	0.134	0.139	0.140	−0.442**	194
		[0.187]	[0.188]	[0.183]	[0.174]	[0.057]	
Fear, EATQ	2.701	−0.063	−0.063	−0.101	−0.074	−0.217*	194
		[0.069]	[0.069]	[0.083]	[0.080]	[0.107]	
Frustration, EATQ	2.996	0.060	0.059	−0.018	−0.001	−0.423*	194
		[0.118]	[0.118]	[0.102]	[0.147]	[0.193]	
Inhibitory control, EATQ	3.426	−0.110*	−0.111*	−0.150	−0.138	0.008	194
		[0.048]	[0.048]	[0.106]	[0.080]	[0.061]	
Growth Mindset	3.436	0.347*	0.345*	0.297	0.316	−0.428	194
		[0.176]	[0.176]	[0.221]	[0.191]	[0.615]	
Reaction time, Stroop	1245	−163.331	−162.391	−203.864	−217.067*	128.509	194
		[115.660]	[116.265]	[129.744]	[90.693]	[92.734]	
Errors, working memory task, 6	4.304	−1.692*	−1.684*	−1.800	−2.063*	0.290	194
		[0.828]	[0.832]	[1.158]	[0.907]	[0.964]	
Errors, working memory task, 8	13.78	−2.066	−2.066	−1.043	−1.315	1.243	194
		[3.093]	[3.101]	[3.833]	[3.324]	[3.148]	
Stickers given – Dictator game	2.605	1.618	1.612	1.787	1.488	−0.251	194
		[1.520]	[1.530]	[1.472]	[1.558]	[0.587]	
Social ties – total	4.270	−0.311***	−0.311***	−0.433***	−0.390***	0.101	194
		[0.078]	[0.079]	[0.087]	[0.086]	[0.151]	
Social ties – strong friendships	1.973	−0.158	−0.16	−0.244	−0.335	0.667	194
		[0.422]	[0.422]	[0.538]	[0.560]	[1.212]	
Social ties – popular	3.459	1.029*	1.026*	0.728	0.749	−0.137	194
		[0.471]	[0.472]	[0.649]	[0.535]	[0.332]	

*Robust standard errors clustered at class level in brackets. SDQ, Strength and Difficulties Questionnaire; EATQ, Early Adolescent Temperament Questionnaire. Controls in model RE-1 include time (March 2020 versus November 2019), treatment (school versus control school) and class dummies. Controls in model RE-2 additionally include month and year of birth dummies. Controls in model RE-3 additionally include: dummy for gender, a dummy for the test taken in the morning; dummy for learning disability, and the interaction between learning disability and time. Controls in model RE-4 include those in other specifications and the baseline value of the dependent variable. Controls in model RE-5 are the same as in model RE-4, and the coefficient shows the interaction with female. The sample size consists of 100 observations at baseline, and of 94 at the follow up. Missing data are imputed.*

***p < 0.001, **p < 0.01, and *p < 0.05.

The first two rows of Table 6 show the results for the Child Outcomes Rating Scale (CORS), measured both as a continuous variable and as a binary indicator of poor well-being; we find no significant change for either measure. When comparing the average values before and after the intervention, the well-being of students in both groups declined, by 3 percent for the continuous variable and 10 percent for the binary indicator: this is possibly due to COVID-19, since the follow-up data was collected a few days before the lockdown.

The coefficients for the High Social Difficulty Score and the Strengths and Difficulty Questionnaire (SDQ) externalizing score are mostly negative across the three specifications, but they do not reach statistical significance. For the temperament measures (EATQ), the results indicate that inhibitory control decreased in the treatment group compared to the control group, however, although stable across the specifications, the coefficient is driven to insignificance when all controls are included. When we look at gender differences, we find that girls in the treatment group have significantly less feelings of aggression, frustration, and fear, suggesting that the intervention may have beneficial effects for their emotional well-being.

The coefficient for growth mindset is positive in the baseline specification, and trending toward significance when all controls are included. In the more controlled specification, we find a significant reduction in reaction time to the Stroop test, which is 17 percent lower in the treatment group. This decrease must be interpreted against an increase in reaction time experienced by the control group between baseline and follow up (see Table 5).^19^ Our results for the Working Memory Task also show a better performance for the 6-task version in the treatment group, which showed 2 fewer errors on average – replicating the results obtained in United Kingdom setting. For the 8-task version, the coefficient is also negative, but imprecisely estimated.

In terms of the social outcomes, there is no significant change in social preferences, as measured by the number of stickers given to another child.^20^ However, the composition of the social network changes significantly for the treatment group: the total number of friends declines by 10 percent after the QT intervention (note that the total number of friends was a 0.5 significantly higher in the treatment group at baseline). Popularity increases significantly, but only in the baseline specification. No other significant changes are found for the other two measures of social networks.^21^

In sum, these findings provide suggestive evidence of impacts within the Irish setting on some outcomes (all results are confirmed in the sample of children with no disabilities), although the sample size limitations imply that these should be confirmed in a fully powered trial.

## Discussion

This study investigated for the first time the feasibility of implementing and evaluating the school-based QT meditation practice in a primary school setting in English-speaking European countries. School-based interventions occur within an ideal social setting to allow students to practice and refine their skills (Taylor et al., 2017) and may offer cost-effective alternatives to out-of-school initiatives, in terms of lower resource requirements and the wider number of children that they serve. The per-child cost of a trained TM teacher delivering QT to a child is £190. As meditation practices within schools are becoming increasingly popular, studies on their implementation and the feasibility of conducting rigorous evaluations of these interventions are acutely needed. The present study provides evidence on engagement with, and feasibility and acceptability of, the QT intervention in two settings, one in the United Kingdom and another one in the Irish school system, using a quasi-experimental design.

In sum, the results suggest that schools and students were willing to engage with QT, as evidenced through high recruitment and retention rates in both settings. In addition, acceptability, as reported by the majority of students, was relatively high in the Irish setting. However, data collection suffered from missing data, and implementation fidelity, although measured using self-reports, varied in the two educational settings. These findings are consistent with the evidence in Emerson et al. (2020) which shows that implementation fidelity is only partially achieved in school-based mindfulness interventions. Indeed, a contribution of this study is to test the implementation of the same program in two settings which operate under different educational systems. Thus, our results indicate that context and setting can play an important role in implementation fidelity, with lower level of fidelity in the United Kingdom setting due to the timing of exams. Our study also contributes to the literature by examining the potential malleability of a wide range of children’s skills to intervention in middle childhood. The analysis on the outcome measures indicates that working memory is the most promising measure, as it seems positively affected by the intervention in both studies. This is consistent with the evidence in Waters et al. (2015), showing that the large majority of mediation studies (73 percent) found statistically significant effects on cognitive functioning. A fully powered randomized controlled trial is required to provide more robust estimates of causal impacts.

In terms of program implementation, we found some differences in compliance across the two settings. The implementation of the program requires a daily commitment by teachers and students. Students are encouraged to meditate for 10–15 min in the morning and in the afternoon every day under the supervision of the schoolteacher. The intervention requires the consistency of the practice by all teachers during the school day. Teachers learn the technique 2 months before the students, to become familiar with the practice and to build the habit of practising it. All teachers are expected to lead the meditation practice in the morning (ideally the first period) and afternoon (ideally the last period) at school. The intervention showed high feasibility in the Irish setting, where teachers consistently maintained the daily practice of QT twice a day for 4 months, essentially embedding the practice into the regular school-day. However, for children in the United Kingdom setting, the practice was interrupted for 2 months, and the teachers reported encountering some difficulty in embedding QT regularly into the school day during the SAT preparation period. These differences may be due to two factors. On the one hand, they might reflect a difference between the two educational systems, with the English system emphasizing maximizing academic achievement, possibly at the expense of broader well-being and personal development. On the other hand, it may be related to the timing of intervention delivery. In Ireland, the intervention was delivered to the students in November, while in the United Kingdom it was delivered after mid-January. Delivering the QT program closer to the beginning of the academic year may have helped to form and establish good habits of meditation practice. If the second factor is more relevant than the first, delivering the intervention earlier in the academic year might allow habits of meditation to be established by the time the SAT exams take place; thus, QT could be used as a tool to cope with the stress of the tests.

While the quasi-experimental design was deemed acceptable by the schools and gives some confidence that a randomized design (e.g., by class) would be feasible in the future, the tests completion rate was quite low, particularly for the tasks placed toward the end of the assessment battery. Since we found no differences in the number of assessments completed by treatment status, this suggests student fatigue with long testing. Thus, it is advisable for future studies to consider a narrower set of outcomes which can be completed in a shorter timeframe, or to allow for a longer assessment time; another possibility would be to have more regular but shorter testing sessions, or other forms of data collection.

We also attempted to assess the acceptability of the intervention, but the low completion rate in the United Kingdom setting cannot provide conclusive evidence. In the Irish setting, where the completion rate was about 88 percent, 76 percent of the students who answered the acceptability question found that the meditation was easy to do, and 70 percent liked it and found it helpful. In both settings, the teachers informally reported that the meditation was easy to implement and that students looked forward to the daily meditation practice. They also said that the practice calmed the students and allowed the teaching to run smoothly after the session. Also, albeit based in a smaller sample, parents in the Irish setting noted that their children became calmer since the beginning of the intervention.

Regarding the initial impacts of the intervention, recall that this demonstration study was not designed or powered to test the effectiveness of QT. Rather, it was designed to assess the feasibility of applying a quasi-experimental design and evaluate which outcomes might be feasible to collect. That said, the results of the outcome analysis provide some suggestive evidence that the intervention may improve certain dimensions of children’s skills. First, in terms of executive function, the QT intervention improved working memory (fewer mistakes made in the task) both in the United Kingdom setting, when compared to a similarly active control condition (the Head Space app), and in the Irish setting, when compared to a non-active control condition. The Reaction Time in the Stroop game, however, improved only in the Irish setting. Second, in terms of social outcomes, there was no significant change in the United Kingdom setting. On the other hand, in the Irish setting, the total number of friends decreased in the treatment group (which nonetheless had more friends at baseline. Third, regarding the well-being outcomes (which were only measured in the Irish setting), the results indicate that girls who participated in the QT practice experienced fewer feelings of aggression, fear, and frustration. Fourth, regarding academic performance (which was only measured in the United Kingdom setting), we did not find significant improvements in English or Mathematics scores between the treated and control groups. In both settings, none of these results retained statistical significance once we accounted for the multiplicity of the hypotheses tested. This result is unsurprising as the studies were not powered to test the effectiveness of the intervention, thus, while many of the treatment effects are of the expected sign, they fail to reach statistical significance. Nonetheless, these findings are promising and would benefit from thorough testing in a full-scale randomized trial.

### Study Strengths and Limitations

This study presents some strengths as well as some limitations. In terms of strengths, the QT intervention was delivered in a standardized way across both countries and educational systems, it was implemented by schoolteachers and involved all students in the class (if a child did not participate, he/she could sit quietly during QT). This limits concerns regarding self-selection bias or threats to external validity that arise in contexts where programs are delivered by specialists. Moreover, unlike other meditation strategies, such as Mindfulness Meditation which is taught in 6 to 9 one-hour lessons, once learned, QT is practiced 10–15 min every day twice a day in class. Thus, implementation of QT may be more feasible in busy school settings where there are multiple demands on teacher’s time. Additionally, our study is one of the few to compare Transcendental Meditation with an equally novel and popular active control condition such as Head Space (in the United Kingdom setting), rather than a passive no-treatment condition or waitlist. While there is some suggestive evidence that TM may be more beneficial for working memory, further evaluation is required.

This study also presents some limitations. One limitation concerns the measurement of compliance with the treatment which was based on self-reports of daily practice to the QT practitioners, rather than independent observations of compliance. Hence, more objective measures of compliance are a priority for future studies. The DLF has recently developed an App where teachers can sign in and track their meditation practices, allowing, if used properly, to measure compliance with the treatment and its intensity.

A second limitation concerns the study design which was based a staggered implementation design using comparison classrooms/schools rather than random assignment. One consequence of this strategy is that not all demographic characteristics and outcomes were balanced at the baseline. Another possible consequence of this quasi-experimental design is that unobserved characteristics may have affected the treatment efficacy instead of, or in addition to, the QT intervention.

Finally, another limitation concerns the follow-ups. On the one hand, we could not follow up students in the United Kingdom setting for longer than the academic year, since they moved to secondary schools at the end of sixth grade; on the other hand, in the Irish setting, COVID-19 and the resulting lockdown precluded the collection of additional follow-up data. To overcome this limitation, we have also explored the option of delivering the intervention in secondary schools, however, it is difficult to implement QT regularly during the school day in secondary schools in England, due to the very intense schedule teachers face and the high number of teachers that students are exposed to. As students in primary schools are exposed to fewer teachers, the implementation of QT in primary schools guarantees greater consistency.

## Conclusion

Our results suggest that QT may be a viable and acceptable practice in a school-based setting when implemented on a regular basis by trained teachers. The estimates of the treatment effects (the interaction terms between the treatment dummy and the follow-up dummy) estimated in the two pilot studies in the United Kingdom and in Ireland will be used to power a larger-scale RCT of the intervention to explore the impact of implementing QT in a wider and more diverse set of primary schools.

## Data Availability Statement

The datasets presented in this article are not readily available because participants of this study did not agree for their data to be shared publicly. Requests to access the datasets should be directed to the corresponding author.

## Ethics Statement

The studies involving human participants were reviewed and approved by Human Research Ethics Committee – Humanities, University College Dublin; UCL IOE Research Ethics Committee, UCL Institute of Education. Written informed consent to participate in this study was provided by the participants’ legal guardian/next of kin.

## Author Contributions

GC, PF, and VO contributed to funding and the conceptualization of the project. OD and VO contributed to the ethics application and wrote a first draft of the manuscript, that was then reviewed by GC and PF. VO and GC managed the project, collected the data, and analyzed them. All authors contributed to the article and approved the submitted version.

## Conflict of Interest

The authors declare that the research was conducted in the absence of any commercial or financial relationships that could be construed as a potential conflict of interest.

## Publisher’s Note

All claims expressed in this article are solely those of the authors and do not necessarily represent those of their affiliated organizations, or those of the publisher, the editors and the reviewers. Any product that may be evaluated in this article, or claim that may be made by its manufacturer, is not guaranteed or endorsed by the publisher.

## Footnotes

1 Mental health among young people has deteriorated dramatically in the first month of the coronavirus pandemic in the United Kingdom (Pierce et al., 2020).

2 In 2019, up to 370 British schools introduced mindfulness into their curriculum as part of a trial issued by the Government to tackle young adults’ mental health disorders.

3 Testimonials of the intervention can be found at https://www.davidlynchfoundation.org.uk/schools.html.

4 A description of the study can be found at https://europe-project.org/the-project/.

5 The school is a non-selective school which uses geographical proximity of the students’ residence as the main criterion for enrollment rather than academic achievement. It was rated as Outstanding in every dimension in the latest assessment by the Office for Standards in Education.

6 The DLF offered the delivery of QT to fifth grade students in the academic year 2019–2020. However, due to planning delays and the COVID-19 outbreak, they did not receive the intervention.

7 Both are mixed gender, small state schools with a catholic ethos. Neither school is classified as a designated disadvantaged school and the average class size is 10 and 11 students respectively.

8 Four percent of children in the both settings showed a high error rate. We explored whether error rates where significantly different between children in the treatment and the control group: the coefficient is close to zero and not significant in both studies so we chose not to report this outcome. Children with a high error rate are excluded from the reaction time analysis.

9 Observable characteristics were not significantly different between students who completed the assessment at baseline and those who did not, except for the proportion of students from an ethnically mixed background, which is higher among students who did not complete the assessment at baseline.

10 These results are robust to using permutation tests, which is warranted given the small sample size.

11 Source: Department for Education “Schools, pupils and their characteristics: January 2018.”

12 The treatment school capacity is 279 students, and the control school capacity is 255 students.

13 Data on students’ learning disabilities was only available at the end of the academic year, so we could not account for this at the beginning of the intervention.

14 We also include a control for disability status and the interaction between time and disability status to capture any difference in the outcomes which might be associated with the learning disability.

15 Recall that the children completed these questions 1 week after seating the SAT, which might have implied higher fatigue on their side.

16 Most literature [for example, Rubin (1987) and van Buuren et al. (1999)] suggests that estimating five datasets should be sufficient to obtain valid inference.

17 This holds also accounting for the age differences.

18 Testing many hypotheses may give rise to concerns about multiple inference. In this context, the probability that we incorrectly reject at least one null hypothesis is greater than the significance level used for each individual hypothesis test. We address this multiple inference concern by controlling for the familywise error rate, i.e., the probability of incorrectly rejecting one or more null hypotheses belonging to a family of hypotheses. To control for the familywise error rate, we define five mutually exclusive families of hypotheses that encompass all our outcome variables. Each family contains all variables belonging to one of the three outcome domains: executive functions, academic achievement, and social measures. When testing multiple hypotheses using the random effects panel regression, we calculate family adjusted *p*-values based on 1,000 bootstraps using the procedure of Romano-Wolf (Clarke et al., 2020). The adjusted *p*-values computed with this procedure indicate that none of the results is statistically significant.

19 The follow-up testing was conducted on the same day in both schools, but at different times of the day in each class. We control for this in the analysis, in model RE-2 and RE-3.

20 The social component of the SDQ is positive but it is not significantly affected by the intervention.

21 As for the United Kingdom setting, we address multiple inference concerns by controlling for the familywise error rate. We define four mutually exclusive families of hypotheses that encompass all our outcome variables: well-being and growth mindset, socio-emotional skills, executive functions, and social measures. When testing multiple hypotheses, we calculate family adjusted *p*-values based on 1,000 bootstraps using the procedure of Romano-Wolf (Clarke et al., 2020). The adjusted *p*-values computed with this procedure indicate that none of the results is statistically significant.
